# Reversible heart rhythm complexity impairment in patients with primary aldosteronism

**DOI:** 10.1038/srep11249

**Published:** 2015-08-18

**Authors:** Yen-Hung Lin, Vin-Cent Wu, Men-Tzung Lo, Xue-Ming Wu, Chi-Sheng Hung, Kwan-Dun Wu, Chen Lin, Yi-Lwun Ho, Michael Stowasser, Chung-Kang Peng

**Affiliations:** 1 Department of internal medicine, National Taiwan University Hospital and National Taiwan University College of Medicine, Taipei, Taiwan; 2 Center for Dynamical Biomarkers and Translational Medicine, National Central University, Chungli, Taiwan; 3 Department of Internal Medicine, Taoyuan General Hospital, Taoyuan, Taiwan; 4 Endocrine Hypertension Research Center, University of Queensland School of Medicine, Greenslopes and Princess Alexandra Hospitals, Brisbane, Australia; 5 Division of Interdisciplinary Medicine and Biotechnology, Beth Israel Deaconess Medical Center/Harvard Medical School, Boston, Massachusetts, USA

## Abstract

Excess aldosterone secretion in patients with primary aldosteronism (PA) impairs their cardiovascular system. Heart rhythm complexity analysis, derived from heart rate variability (HRV), is a powerful tool to quantify the complex regulatory dynamics of human physiology. We prospectively analyzed 20 patients with aldosterone producing adenoma (APA) that underwent adrenalectomy and 25 patients with essential hypertension (EH). The heart rate data were analyzed by conventional HRV and heart rhythm complexity analysis including detrended fluctuation analysis (DFA) and multiscale entropy (MSE). We found APA patients had significantly decreased DFAα2 on DFA analysis and decreased area 1–5, area 6–15, and area 6–20 on MSE analysis (all p < 0.05). Area 1–5, area 6–15, area 6–20 in the MSE study correlated significantly with log-transformed renin activity and log-transformed aldosterone-renin ratio (all p < = 0.01). The conventional HRV parameters were comparable between PA and EH patients. After adrenalectomy, all the altered DFA and MSE parameters improved significantly (all p < 0.05). The conventional HRV parameters did not change. Our result suggested that heart rhythm complexity is impaired in APA patients and this is at least partially reversed by adrenalectomy.

Primary aldosteronism (PA) is characterized by an inappropriate production of aldosterone and affects 5–13% of hypertensive patients^1^. Recent studies indicate that PA is as the most frequent cause of secondary hypertension^1^.

Patients with PA have a higher incidence of cardiovascular events than patients with essential hypertension (EH) matched for hypertension severity^2^. PA patients also have a higher incidence of altered cardiac structure, such as left ventricular hypertrophy and cardiac fibrosis than EH patients, which may due to the high plasma aldosterone levels in these patients^3^. As well as altering cardiac structure, excess aldosterone also increases vascular stiffiness^4^. After removal of aldosterone excess by adrenalectomy or blockade of aldosterone action by mineralocorticoid receptor antagonists, the altered structure of the cardiovascular system recover^4^,^5^.

Aldosterone also influences the autonomic system. In animal studies, infusion of aldosterone into cerebral ventricles increases sympathetic nerve activity (SNA) and blood pressure (BP), effects which are antagonized by mineralocorticoid antagonist administration^6^ . In clinical studies, the reported effects of aldosterone excess on the autonomic system have been inconsistent. Three muscle SNA studies were measured by intraneural microelectrodes in PA patients gave conflicting results^7^,^8^,^9^.

Analysis of the variation of heart rate oscillation, mostly known as heart rate variability (HRV), is commonly used to assess the alteration of autonomic function in human studies due to its simple, noninvasive, and inexpensive approach^10^. Traditional linear analysis of HRV is also used as a tool to evaluate the autonomic system, and especially high frequency power spectrum analysis to evaluate parasympathetic function^10^. HRV has been commonly applied to predict outcome in patients with cardiovascular disease^11^. In recent years, newer methods based on nonlinear and nonstationary signal modeling have been developed and successfully applied^12^. The concept of heart rhythm complexity analysis by nonlinear methods including detrended fractal analysis (DFA) or multiscale entropy (MSE) is based on the assumption that a healthy system exhibits a meaningful complex control over different time scales to maintain operation in ever-changing environment^13^,^14^. Conversely, decreased complexity of heart rate dynamics has been demonstrated in patients with diseases states, such as heart failure, stroke, sepsis, and critical illness requiring extracorporeal life support^15^,^16^,^17^,^18^. Compared to traditional HRV parameter based on linear methodology, heart rhythm complexity analysis showed better predictive power for prognosis in patients with cardiovascular disease^15^,^19^.

Whether aldosterone excess affects heart rhythm complexity is unclear and formed the basis of the current study.

## Results

### Patients

Twenty patients (9 men) with aldosterone producing adenoma (APA) undergoing adrenalectomy and 25 patients with EH were enrolled. The clinical data are shown in Table 1. Patients with APA had significantly higher plasma aldosterone concentration (PAC), higher left ventricular mass index, higher plasma aldosterone-to-renin activity ratio (ARR), lower serum potassium levels, and lower plasma renin activity (PRA) than patients with EH. Regarding medication usage, a significantly higher percentage of APA patients received α-blocker and spironolactone treatment, and lower percentage received angiotensin receptor blocker (ARB) treatment.

### Holter data

**Figure 1:**
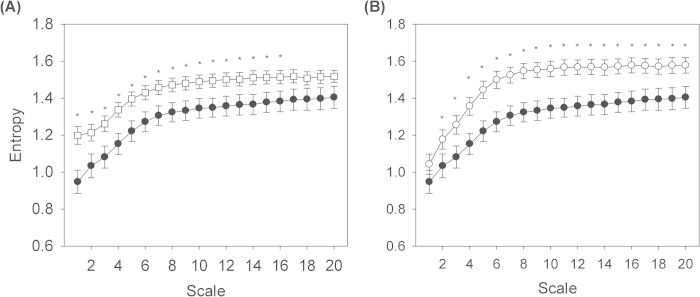
The entropy over different time scales. **a**) The square open symbols represented the entropy of patients with EH, and the black solid dots the entropy of APA patients before surgery. **b**) The black solid dots represented the entropy of APA patients before surgery, and the open circles the entropy of APA patients after surgery. *p < 0.05.

For all (Table 2) linear Holter parameters, there were no differences between the APA and EH groups (all p > 0.05). For non-linear parameters, the APA group had significantly higher DFAα2 (1.19 ± 0.09 vs. 1.13 ± 0.10, p = 0.046), lower area 1–5 (5.4 ± 1.3 v.s. 6.4 ± 1.0, p = 0.006), lower area 6–15 (13.4 ± 2.4 vs. 14.9 ± 1.8, p = 0.023) and lower area 6–20 (20.4 ± 3.6 vs. 22.4 ± 2.7, p = 0.036) of the MSE parameters than EH group. The entropy over different time scales in APA and EH groups was shown in Fig. 1a. The MSE parameters, including area 1–5, area 6–16, and area 6–20 significantly correlated with Log PRA and Log ARR (all p < 0.01) (Table 3). Serum potassium levels also significantly correlated with area 6–16 and area 6–20 (both p < 0.05).

In multi-variate regression analysis, Log PRA was an independent factor associated with area 6–15 (β = 0.803, p = 0.007) and area 6–20 (β = 1.158, p = 0.010) in the final models (excluded variables: serum potassium levels and Log ARR in both models).

### Post-adrenalectomy follow-up

By one year after adrenalectomy, the number of anti-hypertensive medications, log PAC, log ARR, and left ventricular mass index had significantly decreased while PRA and serum K level had increased (Table 4). Eleven out of 20 patients were cured of hypertension.

Repeat Holter study (Table 5) did not reveal any significant change in linear HRV parameters post-operatively. In contrast, DFA*α*2 decreased significantly (from 1.19 ± 0.09 to 1.13 ± 0.13, p = 0.043). The entropy over different time scales in pre- and post-operative APA patients was shown in Fig. 1b. Within the MSE parameters, slope 5 (from 0.067 ± 0.055 to 0.098 ± 0.051, p = 0.035), area 1–5 (from 5.4 ± 1.3 to 6.3 ± 1.1, p = 0.007), area 6–15 (from 13.4 ± 2.4 to 15.5 ± 1.8, p = 0.001), and area 6–20 (from 20.4 ± 3.6 to 23.4 ± 2.7, p = 0.003) increased significantly.

## Discussion

The major findings of this study were 1) APA patients had worse heart rhythm complexity than EH patients, and this was independent of BP; 2) In the correlation study regarding all participants, the parameters of heart rhythm complexity correlated with Log PRA and Log ARR, but not blood pressure or altered cardiac structure; 3) the impaired heart rhythm complexity improved after adrenalectomy.

The presented study is the first study to show the adverse effects of aldosterone on heart rhythm complexity, which quantifies the complex regulatory dynamics of human physiology^20^. HRV is commonly used to assess autonomic function and risk stratification of patients with cardiovascular disease^11^,^21^. Compared to traditional linear HRV parameters, nonlinear metrics (including DFA and MSE) showed better predictive power for clinical outcomes in heart failure patients and in an experimental sepsis model^15^,^19^,^22^. Recently, the MSE method, specifically developed to treat heterogeneous complexity, was shown to extend the traditional entropy algorithm to quantify the information richness over multiple time scales in physiological systems^20^. This complex structure will “break down” in diseased patients, such as those with heart failure or with critical illness, and may be further affected in those with poor prognosis^15^,^18^. In our previous study, MSE provided the best prognostic prediction in patients with heart failure^15^. MSE also predicted outcome of severe traumatic patients requiring intensive care unit admission across the diverse spectrum of traumatic injury^23^, the neurological outcome after stroke^16^, the clinical consequences of sepsis^17^, and outcome of patients with critical illness receiving extracorporeal life support^18^. In the present study, PA patients had worse heart rhythm complexity than EH patients, suggesting that aldosterone excess impaired the complex regulatory dynamics of human physiology. Furthermore, there were significant associations between heart rhythm complexity and log PRA and log ARR, but not BP or cardiac structure in the correlation study regarding all participants. This implied a direct association between aldosterone and heart rhythm complexity. Moreover, after adrenalectomy, the impaired heart rhythm complexity improved, suggesting that the impairment is at least partially reversible.

In contrast to the significant differences of values in non-linear parameters between PA and EH patients, the values of traditional HRV parameters (such as time and frequency domain parameters) were comparable in both groups in the current study. This also applied when comparing the pre and post-operative data of APA patients. As mentioned earlier, traditional HRV parameters are commonly used to evaluate the autonomic system, and particularly high frequency power spectrum analysis to evaluate parasympathetic function^10^. Data from the current study did not provide further evidence to support PA patients having worse autonomic function than EH patients.

The relation between aldosterone and the autonomic system is complex. In animal studies, excess of aldosterone is associated with autonomic dysfunction. It was found that there are mineralocorticoid receptors near the hippocampus and regulated by mineralocorticoids and glucocorticoids^24^. Further, aldosterone infusion into cerebral ventricles increased SNA and BP, an effect which was antagonized by administration of a mineralocorticoid antagonist^6^. In another animal model, the increased BP induced by aldosterone was blocked by chemical sympathectomy^25^. In the elderly hypertensive population, the mineralocorticoid antagonist spironolactone decreased norepinephrine levels^26^. The evidence suggests a close relationship between aldosterone and the autonomic system.

Whether PA patients have impaired autonomic function is controversial. Acute infusion of aldosterone in healthy volunteers increased the standard deviation of RR intervals and total power, and was associated with a trend towards increased time domain HRV parameters^27^. However, in that study, basal muscle SNA, BP and heart rate remained unaffected by aldosterone administration. This suggests that, acutely, aldosterone infusion tends to increase cardiac vagal activity and has no effect on sympathetic activity. PA patients have lower sympathetic vasomotor tone than patients with EH^28^. Several studies have evaluated muscle SNA in patients with PA^7^,^8^. In the study by Kontak *et al.*, PA patients had similar muscle SNA to EH patients^7^. Both PA and EH patients had higher muscle SNA than normotensive subjects. However, the muscle SNA decreased significantly after adrenalectomy, accompanied by a decrease in BP^7^. Whether removal of aldosterone excess or decrease of BP contributed to this improvement is unclear. In another study by Miyajima *et al.*, PA patients had lower muscle SNA compared to EH patients^8^. In a third study by Matsukawa *et al.*^9^, PA patients have lower muscle SNA compared to normotensive subjects. Interestingly, the muscle SNA was significantly elevated after unilateral adrenalectomy in PA patients. It was in contrast to the study by Kontak *et al.*^7^. Although different race and other inter-individual differences may have contributed to this disparity, it may also reflect the fact that regulation of sympathetic activity in PA patients is complex and influenced by factors other than aldosterone itself.

The relationship between aldosterone and parasympathetic activity is also complex and conflicting data exist. Acute infusion of aldosterone in healthy volunteers increased cardiac vagal activity in one HRV study^27^. Aliskirin, which reduces activity of the renin-angiotensin-aldosterone system via direct inhibition of renin, increased parasympathetic function as measured by two cardiovascular autonomic reflex tests^29^. However, the high frequency domain of HRV, an index of cardiac parasympathetic tone did not change^29^. Studies assessing parasympathetic function in PA are quite few. To the best of our knowledge, there are only two studies from the same group dealing with this issue. In the first, the baroreflex sensitivity of PA patients was found to be similar to age-matched healthy subjects. In contrast, the baroreflex sensitivity was impaired in EH patients^30^. In the second study from the same group, PA patients showed similar high frequency in HRV compared to normotensive subjects, but high frequency in HRV was decrease in patients with EH^28^. These data suggest that, although hypertensive, PA patients have intact parasympathetic activity. Against this notion, however, was the observation that the high frequency value decreased after adrenalectomy in the PA patients^28^.

In this study, we only enrolled APA patients that underwent adrenalectomy, but not patients with bilateral adrenal hyperplasia. Adrenalectomy is the treatment of choice for APA patients. In contrast, medical treatment with spironolactone is the treatment of choice in patients with bilateral adrenal hyperplasia. Compared to medical treatment with spironolactone, adrenalectomy decreases left ventricular mass more quickly^31^. In another study, only adrenalectomy but not spironolactone improved arterial stiffness in PA patients (average follow-up period: one year)^32^. This evidence implies that adrenalectomy is a more effective method to reverse the effects of aldosterone on cardiovascular system. Therefore, we enrolled APA patients that underwent adrenalectomy as the study group to enhance the difference between pre- and post-treatment.

One advantage of this study is that the BP and the number of antihypertensive medications was comparable between APA and EH groups. It is common that PA patients have significantly higher BP than EH patients^33^. In our previous studies, APA patients also had significantly higher BP than EH patients^4^,^5^. However, any BP difference (for example, if APA patients had significantly higher BP than EH patients in this study) would make the interpretation of results more difficult and complex. Although the correlations between HRV parameters and BP were not significant, we still could not exclude a possible confounding effect from a difference in BP. Thankfully, the BP difference was not significant between APA and EH groups in this study, which made the interpretation easier.

Our study has several limitations. First, the study population is small. Further large prospective studies are needed to confirm the results. Second, we did not measure muscle SNA to evaluate sympathetic activity. However, the main purpose of this study was to evaluate the influence of aldosterone on heart rhythm complexity rather than sympathetic activity per se.

In conclusion, APA patients had impaired heart rhythm complexity compared to EH patients, and this was independent of BP. The impaired heart rhythm complexity improved after adrenalectomy.

## Methods

### Patients

This prospective study enrolled 20 patients diagnosed with unilateral APA and who underwent adrenalectomy during the period from December 2006 to October 2009. The patients were evaluated and registered in the Taiwan Primary Aldosteronism Investigation (TAIPAI) database. The database was constructed for quality assurance in two medical centers (National Taiwan University Hospital (NTUH), Taipei; Taipei Medical University Hospital, Taipei), three metropolitan hospitals (Cardinal Tien Hospital, New Taipei City; Taipei Tzu Chi Hospital, New Taipei City; Yun-Lin Branch of NTUH, Douliou City), and two local hospitals (Hsin-Chu Branch of NTUH, Hsin-Chu City; Zhongxing Branch of Taipei City Hospital, Taipei)^34^,^35^,^36^. In addition, another 25 patients with EH were enrolled as the control group. Medical history including demography and medication was carefully recorded. Biochemical parameters were measured at the first evaluation of these patients in National Taiwan University Hospital. PRA was measured as the generation of angiotensin-I *in vitro* using a commercially available radioimmunoassay kit (Cisbio, Bedford, MA); PAC was measured by radioimmunoassay with commercial kits (Aldosterone Maia Kit; Adaltis Italia, Bologna, Italy). All antihypertensive medications were discontinued for at least 21 days before measuring plasma PRA and PAC levels. Diltiazem and/or doxazosin were administered for control of marked high blood pressure when required. All APA patients underwent 24-h ambulatory ECG Holter recording (MyECG E3-80, Mircostar Company, Taipei) within 3 months before operation and one year after operation. EH patients also underwent 24-h ambulatory ECG Holter recording at the time of enrollment. The ECG signals were sampled at 250 Hz and stored in secure digital memory card for offline analysis on a microcomputer. This study was approved by the Institutional Review Board of National Taiwan University Hospital, and all subjects gave informed consent in written form including for the storage of their information in the hospital database and usage for research. The methods in the study were carried out in accordance with the approved guidelines.

### Data Pre-Processing

Each digitalized 24-hour ECG data was annotated by an automatic algorithm then carefully inspected and corrected by the technicians for extracting the RR intervals and the ectopic beats were interpolated by its adjacent RR intervals. A four-hour length of RR intervals within daytime (between 9 AM–5 PM) was selected for analysis in order to avoid the confounding effects that may occur due to sleep or diurnal rhythm^37^. Only the RR series of subjects in whom qualified normal sinus beats made up more than 80% of the recording were included for further analysis.

### Time and frequency domain analysis

The standard deviation of normal RR intervals, the percentage of the absolute change in consecutive normal RR interval exceeds 50 ms, and percentage of the absolute change in consecutive normal RR interval exceeds 20 ms were calculated to represent the total variance and vagal modulation of heart rate. The spectrum analyses were carried out according to the recommendations from the European Society of Cardiology and the North American Society of Pacing Electrophysiology^10^. Instead of calculating the spectrum in overall length, we divided the data into 16 segments and Fourier transformation was performed individually to avoid the influence of external nonstationarity. The spectral density of each frequency band- high frequency (0.15–0.4 Hz), low frequency (0.04–0.15 Hz), and very low frequency (0.003–0.04 Hz) were computed by averaging the absolute powers (msec^2^) in separated segments.

### Nonlinear methods

Nonlinear analysis enables researchers to probe the fundamental characteristics of the signals. We apply two methods (DFA and MSE) for their ability to evaluate the underling properties of the signals hidden beneath the seemingly chaotic dynamics^20^,^38^.

### DFA analysis

DFA is a modified root-mean-square analysis that is used to evaluate the fractal correlation (a time-invariant property) beneath the heart rate fluctuation originated from the well-interacted regulatory mechanisms^38^. First, it eliminates the environmental inferences by removing the linear-fitted “local” trend over different time scales in an integrated time series. Then, the root-mean-square fluctuation of this integrated and detrended time series is calculated. This procedure is repeated in different time scales and then the slope of the curve (α exponent) can be computed on the log-log plot of fluctuation versus box size which indicates the fractal correlation property of the time series.

Since the heart rate oscillation over short timescale is predominated by respiratory sinus arrhythmia, a crossover phenomenon of the α exponent in heart rate dynamics between short (4–11 beats) and long (11–64 beats) time scales has been proposed to provide better understanding of the fractal correlation property in a physiological system^38^. The short-term (α1) as well as long-term (α2) fractal correlation were calculated.

### MSE analysis

**Figure 2:**
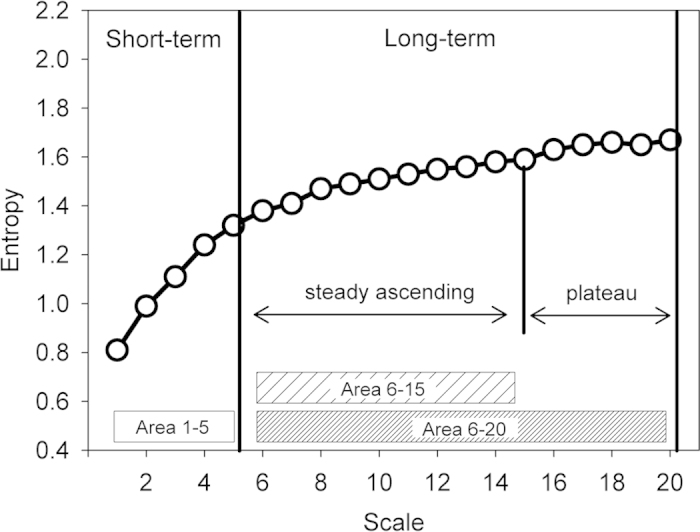
Quantification of MSE: The summation of the entropy over different scales can quantify the complexity over certain time scales. Four parameters of the MSE were assessed. The first two of these were the linear-fitted slope between scales 1–5 (slope 5); the area under curve between scale 1–5 (area 1–5) to represent complexity between short scales. For longer scales, the common profile of entropy gradually increases as the time scale elongates and reaches a plateau around scale 15 where information richness can be accumulated rapidly if the system can respond well. We therefore calculated both the area under curve between scales 6–15 (area 6–15) and 6–20 (area 6–20) to represent complexity between long scales.

In contrast to simply using a single time scale to estimate the predictability of a time series, MSE assesses the complex structure of the physiological signals in different time scales. It comprises two steps: 1) coarse-graining the signals into different time scales; 2) quantifying the degree of predictability in each coarse-grained time series by using sample entropy^39^. The calculated entropy can be represented as a function of scale provides the meaningful information richness embedded in different time scales. It has been shown that different features of small and large scales in different groups of subjects may assist the clinical categorization^20^. Therefore, four different parameters were calculated from the MSE profile: the summations of entropy values of scale 1–5 (area 1–5), scale 6–15 (area 6–15), or scale 6–20 (area 6–20) which quantify the complexity exhibited in short and long time scales, respectively; and the linear-fitted slope of scale 1–5 (slope 5) was also calculated to characterize the behavior of short-scale (Fig. 2)^20^.

In order to avoid an unwanted effect of external nonstationarity which may compromise the entropy-based analysis, we use the empirical mode decomposition method which is based on Hilbert-Huang transform to detrend the original R-R interval signals^40^. The data was subsequently evaluated by the MSE analysis after processing. Instead of removing trend with *a priori* mathematical formulas such as polynomial or linear functions, the empirical mode decomposition algorithm could better approximate the hidden trend in complex time series^40^,^41^,^42^.

### Diagnosis of APA

APA was diagnosed on the basis on the following four conditions: (1) autonomous excess aldosterone production evidenced with an ARR > 35, a TAIPAI score larger than 60%^43^, and post-saline loading PAC > 10 ng/dl; (2) adenoma evidenced with a computed tomography (CT) scan on pre-operative evaluation; (3) lateralization of aldosterone secretion at adrenal venous sampling or during dexamethasone suppression NP-59 SPECT/CT^44^; (4) pathologically proven adenoma after adrenalectomy for those undergoing surgery, and subsequent cure or improvement of hypertension control with correction of hypokalemia and normalization of PAC and PRA^34^,^45^. Dexamethasone suppression NP-59 SPECT/CT was used in patients who were at risk of contrast nephropathy, had inconclusive results from adrenal venous sampling or had discordant lateralization between imaging studies (such as CT) and adrenal venous sampling.

### Echocardiography

A Hewlett-Packard Sonos 5500 ultrasound system equipped with a S3 transducer was used. Echocardiography including two-dimensional, M-mode and Doppler ultrasound recordings were performed. Left ventricular dimension, interventricular septum and posterior wall thicknesses, and left ventricular ejection fraction (M-mode) were measured via a parasternal long axis view. Left ventricular mass index was calculated according to the method of Devereux *et al.*^46^.

### Post-adrenalectomy follow-up

Repeat serum biochemistry, PAC, PRA, 24-h ambulatory ECG Holter recording and echocardiography were performed one year after adrenalectomy. Hypertension was considered cured if the blood pressure decreased to 140/90 mm Hg or less after adrenalectomy, and anti-hypertensive medications were not required. These criteria had to be met one year post adrenalectomy^31^. Patients who were cured within one year but later developed hypertension were still classified as cured.

### Statistical analysis

Data were expressed as mean ± SD. Comparisons for continuous data between APA and EH patients were made using the t test. Differences between proportions were assessed by chi-square or Fisher exact test. Comparisons between pre-operative and post-operative parameters were made using paired t test. The Pearson’s correlation test was used to analyze the association among heart rhythm complexity parameters and its determinants. Data of PAC, PRA, and ARR were log-transformed before the correlation study due to non-normality which was determined by the Kolmogorov-Smirnov test. Before further analysis, the log-transformed data were tested again to assure the normality of distribution. Significant determinants in the Pearson’s correlation test (p < 0.05) were then tested by a multivariate linear regression test with stepwise subset selection to identify independent factors to predict MSE parameters. A value of p < 0.05 was considered to indicate statistical significance.

## Additional Information

**How to cite this article**: Lin, Y.-H. *et al.* Reversible heart rhythm complexity impairment in patients with primary aldosteronism. *Sci. Rep.* **5**, 11249; doi: 10.1038/srep11249 (2015).

## Figures and Tables

**Table 1:** Clinical data of patients at baseline.

Patient characteristics	APA (n = 20)	EH (n = 25)	P
Age, years	45 ± 9	46 ± 11	0.666
Male sex	9 (45)	11 (44)	0.947
History of hypertension, years	5.4 ± 6.6	5.4 ± 4.9	0.982
Body weight, kg	67 ± 13	68 ± 12	0.791
Body height, cm	161 ± 8	164 ± 9	0.270
Creatinine, mg/dL	1.0 ± 0.3	0.9 ± 0.2	0.807
SBP, mmHg	148 ± 13	140 ± 17	0.079
DBP, mmHg	92 ± 11	87 ± 13	0.194
Potassium, mmol/L	3.3 ± 0.8	4.3 ± 0.5	<0.001
PAC, ng/dL	50 ± 36	31 ± 23	0.029
PRA, ng/mL/hr	0.4 ± 0.5	8.9 ± 11.1	0.001
ARR	1932.8 ± 2797.1	23.6 ± 55.8	0.007
Log PAC	1.6 ± 0.3	1.4 ± 0.2	0.006
Log PRA	−0.9 ± 0.9	0.6 ± 0.7	<0.001
Log ARR	2.6 ± 0.9	0.9 ± 0.6	<0.001
LVMI, g/m^2^	135 ± 38	110 ± 27	0.028
LVEF, %	71 ± 7	73 ± 6	0.488
Adenoma diameter on abdominal CT, mm	15.5 ± 6.1	—	—
Adenoma location (left/right side)	16/4	—	—
Number of hypertensive medication	1.7 ± 0.9	1.8 ± 0.8	0.678
Hypertensive Medication			
CCB	13 (65)	15 (60)	0.731
ACEI	0 (0)	2 (8)	0.495
ARB	5 (25)	16 (64)	0.036
Thiazide	3 (15)	1 (4)	0.309
α-blocker	9 (45)	4(16)	0.049
β-blocker	6 (30)	8(32)	0.885
Spironolactone	10 (50)	1 (4)	0.001

Values are mean ± SD or number (percentage). APA = aldosterone producing adenoma; EH = essential hypertension; SBP = systolic blood pressure; DBP = diastolic blood pressure; PAC = plasma aldosterone concentration; PRA = plasma renin activity; ARR = aldosterone-renin ratio; CCB = calcium channel blocker; ACE-I = angiotensin converting enzyme inhibitor; ARB = angiotensin receptor blocker; LVMI = left ventricular mass index; LVEF: left ventricular ejection fraction; CT = computed tomography.

**Table 2:** Holter parameter of patients at baseline.

	APA (n = 20)	EH (n = 25)	P
**Time domain analysis**			
Mean, mm	750 ± 102	739 ± 121	0.746
SDNN, mm	72 ± 18	74 ± 23	0.718
pNN20, mm	20 ± 16	21 ± 13	0.733
pNN50, mm	2.8 ± 4.0	3.2 ± 3.8	0.683
**Frequency domain analysis**			
High frequency	107 ± 115	116 ± 113	0.814
Low frequency	263 ± 154	314 ± 167	0.297
LF/HF ratio	4.0 ± 2.3	4.2 ± 2.9	0.799
Very low frequency	968 ± 376	1018 ± 488	0.711
**Detrended fluctuation analysis**			
α1	1.29 ± 0.19	1.26 ± 0.20	0.613
α2	1.19 ± 0.09	1.13 ± 0.10	0.046
**Multiscale entropy**			
Area 1–5	5.4 ± 1.3	6.4 ± 1.0	0.006
Slope 5	0.067 ± 0.055	0.052 ± 0.058	0.393
Area 6–15	13.4 ± 2.4	14.9 ± 1.8	0.023
Area 6–20	20.4 ± 3.6	22.4 ± 2.7	0.036

Values are mean ± SD. APA = aldosterone producing adenoma; EH = essential hypertension; SDNN = standard deviation of normal RR intervals; pNN20 = percentage of the absolute change in consecutive normal RR interval exceeds 20 ms; pNN50 = percentage of the absolute change in consecutive normal RR interval exceeds 50 ms; LF/HF = the ratio between low and high frequency components.

**Table 3:** Correlation between clinical parameters and non-linear Holter parameters (n = 45).

	DFAα2	*Area 1–5*	*Area 6–15*	*Area 6–20*
Age, years	0.038	−0.048	−0.053	−0.065
Male sex	−0.148	0.044	0.110	0.110
History of hypertension, years	−0.086	0.079	0.091	0.067
Number of hypertensive medication	0.272	−0.052	−0.047	−0.041
Body height, cm	0.162	0.014	−0.090	−0.083
Body weight, kg	−0.128	0.002	0.217	0.208
Log PAC	0.098	−0.190	−0.117	−0.104
Log PRA	−0.177	0.402*	0.395*	0.379*
Log ARR	0.186	−0.416*	−0.393*	−0.376*
Potassium, mmol/L	−0.101	0.181	0.356 #	0.355 #
SBP, mmHg	−0.103	−0.308	−0.014	−0.012
DBP, mmHg	−0.089	−0.015	−0.015	−0.024
LVMI, g/m^2^	0.000	−0.263	−0.200	−0.212
LVEF, %	−0.111	0.087	−0.037	−0.049

Values are correlation coefficients. PAC = plasma aldosterone concentration; PRA = plasma renin activity; ARR = aldosterone-renin ratio; SBP = systolic blood pressure; DBP = diastolic blood pressure; *p < = 0.01; #p < 0.05.

**Table 4:** Clinical parameters before and one year after adrenalectomy (n = 20).

	Before operation	After operation	*P value*
**Clinical parameters**			
SBP, mmHg	148 ± 13	144 ± 16	0.416
DBP, mmHg	92 ± 11	90 ± 12	0.401
Number of hypertensive medication	1.8 ± 1.1	0.7 ± 0.8	<0.001
**Biochemical parameters**			
Potassium, mmol/L	3.3 ± 0.8	4.3 ± 0.5	<0.001
Log PAC	1.6 ± 0.3	1.5 ± 0.2	0.032
Log PRA	−0.9 ± 0.9	−0.1 ± 0.7	0.003
Log ARR	2.6 ± 0.9	1.5 ± 0.7	0.002
**Echocardiographic parameters**			
LVMI, g/m^2^	135 ± 38	113 ± 24	0.004
LVEF, %	71 ± 7	70 ± 9	0.569

Values are mean ± SD. SBP = systolic blood pressure; DBP = diastolic blood pressure; PAC = plasma aldosterone concentration; PRA = plasma renin activity; ARR = aldosterone-renin ratio; LVMI = left ventricular mass index; LVEF: left ventricular ejection fraction.

**Table 5:** Holter parameters before and one year after adrenalectomy (n = 20).

	Before operation	After operation	P
**Time domain analysis**			
Mean, mm	750 ± 102	706 ± 119	0.075
SDNN, mm	72 ± 18	70 ± 19	0.798
pNN20, mm	20 ± 16	16 ± 14	0.208
pNN50, mm	2.8 ± 4.0	2.8 ± 4.0	0.473
**Frequency domain analysis**			
High frequency	107 ± 115	98 ± 120	0.740
Low frequency	263 ± 154	352 ± 357	0.243
LF/HF ratio	4.0 ± 2.3	5.1 ± 3.5	0.188
Very low frequency	968 ± 376	829 ± 373	0.122
**Detrended fluctuation analysis**			
α1	1.29 ± 0.19	1.37 ± 0.17	0.169
α2	1.19 ± 0.09	1.13 ± 0.13	0.043
**Multiscale entropy**			
Area 1–5	5.4 ± 1.3	6.3 ± 1.1	0.007
Slope 5	0.067 ± 0.055	0.098 ± 0.051	0.035
Area 6–15	13.4 ± 2.4	15.5 ± 1.8	0.001
Area 6–20	20.4 ± 3.6	23.4 ± 2.7	0.003

Values are mean ± SD. Values are mean ± SD. APA = aldosterone producing adenoma; EH = essential hypertension; SDNN = standard deviation of normal RR intervals; pNN20 = percentage of absolute differences in normal RR intervals greater than 20 ms; pNN50 = percentage of absolute differences in normal RR intervals greater than 50 ms; LF/HF = low frequency to high frequency.
